# Reviewer acknowledgements

**DOI:** 10.1186/1472-6890-13-2

**Published:** 2013-02-21

**Authors:** Christopher Foote

**Affiliations:** 1 BMC Series, BioMed Central, 236 Grays Inn Road, London, WC1X 8HB, , UK

## Abstract

**Contributing reviewers:**

The editors of BMC Clinical Pathology would like to thank all of the reviewers who have contributed to the journal in volume 12 (2012).

## 

Sharon Akrish

Israel

Simak Ali

UK

Jean-Pierre Allain

UK

Punnya Angadi

India

Yoichi Aoki

Japan

Jan P. A. Baak

Norway

Fatima Baltazar

Portugal

Pedro Baptista

Portugal

Matthew Bates

UK

Luc Bauchet

France

Andrew Beck

USA

Riyad Bendardaf

Finland

Francois-Clement Bidard

France

Ronell Bologna-Molina

Mexico

Theodore Brasky

USA

David Bruns

USA

Lisa Butler

Australia

Daniel Candotti

UK

Jiang Cao

China

Justin Cates

USA

Mu-kuan Chen

Taiwan

Paolo Chieffi

Italy

E. Antonio Chiocca

USA

Daniel Cho

USA

Francois X Claret

USA

Mario D. Cordero

Spain

Paul Cottu

France

Marta Cuadros

Spain

Margaret Currie

New Zealand

Fulvio D'Acquisto

UK

Roger Dodd

USA

Philip Dore

UK

Neil Dufton

UK

Jose Echevarria

Spain

Wafik El-Deiry

USA

Matthias Falk

USA

Charles Fattal

France

Charles French-Constant

UK

Heide Ford

USA

Pallavi Garg

USA

David Gaze

UK

Arkadiusz Gertych

USA

Helenice Gobbi

Brazil

Thomas Greither

Germany

Fabio Grizzi

Italy

Emanuela Guerra

Italy

P Guneri

UK

Junming Guo

China

Xin Guo

Japan

Michael Haas

Germany

Sae-won Han

South Korea

Lise Lotte Hansen

Denmark

Tanja Nicole Hartmann

Austria

Lukas J.A.C. Hawinkels

Netherlands

Zhaohui Huang

China

Nils-Olaf Hübner

Germany

Hiroshi Inagaki

Japan

Jochen Jarausch

Germany

Rzymowska Jolanta

Poland

Ben-Zion Joshua

Israel

Farahnaz Joukar

Iran

Harriette Kahn

Canada

Jia-Horng Kao

Taiwan

Elad Katz

UK

Mahmood Kayani

Pakistan

Jae J Kim

South Korea

Benjamin Kipp

USA

Elena Klenova

UK

Sanjay Kumar Kochar

India

Martin Koebel

Canada

Rituraj Konwar

India

Ja Seung Koo

South Korea

Emin Turkay Korgun

Turkey

Makoto Kunisada

Japan

Roland Kupka

Tanzania

Maciej Kurpisz

Poland

David Kwiatkowski

USA

Athanassios Kyritsis

Greece

Pierre-Jean Lamy

France

Alexander Lazar

USA

Wen-Ying Lee

Taiwan

Herng-Sheng Lee

Taiwan

Sang Kil Lee

South Korea

Marcis Leja

Latvia

Chien-Feng Li

Taiwan

Ying Li

USA

Shu-Chun Lin

Taiwan

Alexander Link

Germany

Michael Linnebacher

Germany

Sally Litherland

USA

Victor Lobanenkov

USA

G. Mike Makrigiorgos

USA

Raphaël Maréchal

Belgium

Renato Mariani-Costantini

Italy

Vicenta Martínez-Sales

Spain

Astuji Matsuyama

Japan

Doris Mayr

Germany

Christopher McEvoy

Australia

Eiji Mekata

Japan

Evangelos Misiakos

Greece

Takuya Moriya

Japan

Loredana Moro

Italy

Hossein Mozdarani

Iran

Maria Aparecida Nagai

Brazil

Keisuke Nakano

Japan

Mark Nellist

Netherlands

Aurelia Noske

Switzerland

Yoshinobu Ohsaki

Japan

Guner Hayri Ozsan

Turkey

Sumanta Pal

USA

Prasit Palittapongarnpim

Thailand

Gopal Pande

India

Maria Luisa Panno

Italy

Jong Park

South Korea

Joel Parker

USA

Elena Pasquale

USA

Carlos Perez-Plasencia

Mexico

John Pettifor

South Africa

Shiv Pillai

USA

Pascal Pineau

France

Marta Pineda

Spain

Daniel PIrici

Romania

Nadege Presneau

UK

Ahmed Rebai

Tunisia

Sandra Rebouissou

France

Donata Rimoldi

Switzerland

Paola Riva

Italy

Darren Roblyer

USA

Ashley Ross

USA

Bo Rueda

USA

Stratigoula Sakellariou

Greece

Kavitha Saravu

India

Nicole Schonrock

Australia

Heidi Schwarzenbach

Germany

Naohiko Seki

Japan

Andrzej Semczuk

Poland

Annabelle Servant-Delmas

France

Toshiki Shimizu

Japan

Cornelis Sier

Netherlands

Katarzyna Sikorska

Poland

Keith Skubitz

USA

Paul Speight

UK

Marc Stemmler

Germany

David Stokoe

USA

Baocun Sun

China

Zoltan Szollosi

Hungary

Tetsuo Takehara

Japan

Turkan Terkivatan

Netherlands

Arafat Tfayli

Lebanon

Kensei Tobinai

Japan

Fumiharu Togo

Japan

David Tougeron

France

Gaffari Turk

Turkey

Nurcan Üçeyler

Germany

Rohit Upadhyay

India

Jose Vassallo

Brazil

Rosane Vianna-Jorge

Brazil

Andrew Vickers

USA

Satish Viswanath

USA

Wei-Lien Wang

USA

Haili Wang

Germany

Scott Weed

USA

Minghua Wu

China

Yaguang Xi

USA

Max Yan

Australia

Yuan Yang

Australia

Andrew Young

USA

Jinming Yu

China

Takeshi Yuasa

Japan

Brad Zacharia

USA

Flora Zagouri

Greece

Bogdan Zalewski

Poland

Shaobo Zhang

USA

Inti Zlobec

Switzerland

Debra Zynger

USA

## Pre-publication history

The pre-publication history for this paper can be accessed here:

http://www.biomedcentral.com/1472-6890/13/2/prepub

